# Stick to Your Teeth!*Note.—This controversy is interesting not only because it indicates a popular interest in dental matters among the medical and dental professions in England, but for the reason that it may very profitably be a topic for professional consideration in America at the present time. It is worth pondering.

**Published:** 1914-10-15

**Authors:** 


### "STICK TO YOUR TEETH!"*

Under the above heading, the *Evening News* of June 16 published a leaderette, which elicited the following reply:

"SIR,—I regret that a paper of such great influence as yours should publish such an article as that entitled 'Stick to your Teeth.' The writer is giving advice upon a vital matter on which a very great amount of work has been done in the last ten years, and on which great authorities take a view exactly opposed to his.

The health of the public is at stake if his advice is unsound, and I am satisfied that it is unsound to lay down as a general principle 'Stick to your teeth.' The following cases (among many others) have come under my care in the past few months:—

THREE CASES.

*Case* 1.—A lady suffering from a deep-seated inflammation of the eyes consulted an oculist some three years ago, who told her that the inflammation was caused by the septic condition of her teeth, and sent her to a dentist, who 'treated these teeth for two years, with the result that she became totally and permanently blind in one eye, and, at the time I saw her, had almost lost the sight of the other.

Immediate extraction of all the teeth has saved this eye; she has now almost perfect sight in this one eye. Had the teeth been extracted three years ago, as the oculist advised, she would have saved the sight of the other.

*Case* 2.—A gentleman suffering from stiffness of the joints of his hands and neck was told by two specialists that he was suffering from rheumatoid arthritis, and that the disease was quite incurable; that all his joints would eventually become permanently fixed, and that nothing could be done for him!

*NOTE.—This controversy is interesting not only because it indicates a popular interest in dental matters among the medical and dental professions in England, but for the reason that it may very profitably be a topic for professional consideration in America at the present time. It is worth pondering.

A living death, truly! Think what it means—unable to walk or write, or even move at all. Immediate extraction of all the teeth has cured him completely. He is now as active as he ever was.

*Case* 3.—A woman was seen in a comatose (unconscious) condition. She was thin, emaciated, picking aimlessly at the bed-clothes, gazing up at the ceiling with a fixed sightless stare. She was obviously going to die in a very few hours.

Ten days after the extraction of her teeth she was sitting up in bed eating fish! She left the hospital quite well four weeks after the extraction.

These three people were all suffering from septic poisoning from their teeth. Retention of these teeth, as advised by you, would have meant blindness in the first case, a fixture of all the joints in the second, and death in the third.

An eye for an eye, a tooth for a tooth was the ancient law, but medical science objects to sacrifice an eye for a tooth, a joint for a tooth, a life for a tooth. Experience has shown that in many cases treatment has to be drastic and prompt.

Your article may in the near future lead to very many other evils beside blindness, and even to death itself, should your advice, 'Stick to your teeth,' be followed by some of your readers."

*D.P.H., L.R.C.P. Lond., M.R.C.S., L.D.S.Eng.*

---

[Our correspondent is as hasty in his conclusions from an article as are his professional brethren who go in for what we called "the clean sweep". We acknowledge quite fairly that there are cases in which extraction is the only course, but as regards the general principle, "Stick to your teeth " we will only say that we gave the advice on the authority of one of the best known dentists in London—Ed., *E. N.*]"

---

In further correspondence, Mr. H. U. Oliver, L.D.S., of London, writes:—

As a result of over thirty years' experience, it is, in my opinion, no less a criminal practice to extract a tooth because it is in an ulcerated or broken down condition than it would be to remove an eye to get rid of a cataract. Yet we are continually hearing of poor unfortunates who have submitted to the extraction of all their teeth to be cured of a gum disease.

Your recent correspondent, D.P.H., L.R.C.P. Lond.,M.R.C.S., L.D.S., Eng.,says that it is unsound to lay down as a general principle "Stick to your teeth," and he regrets that your paper should publish such a heresy. There are a few intelligent dentists, left however, who regard it as a healthy revulsion of feeling, and I am rejoiced to think that this view is shared by the well-known dentist you refer to as your authority.

In the three cases given by your correspondent I notice that his treatment in each was "immediate extraction of *all* the teeth." In isolated cases this may be occasionally necessary, but there is more than a suspicion that when this is done good ones are sacrificed with the bad.

We have been protesting for years against the malpractices of quack dentists, and have viewed with horror the wrecked mouths of the poor deluded people who have been induced to part with all their teeth to make way for wretched substitutes. We have deplored that nothing can be done to suppress these pests of society.

Now, forsooth, we must infer, upon the authority of D.P.H., L.R.C.P.Lond., M.R.C.S., L.D.S.Eng., that in reality these enterprising gentry have been ministering angels in disguise, so "unsound" is it to lay down the principle of "Stick to your teeth."

---

Turning to the Medical Press, the letter of Dr. Goodhart to the *Lancet*—which we reproduced a month ago—has evoked an interesting correspondence. Writing to our contemporary of August 1, Mr. Sidney Spokes says:—

It is, of course, recognized that in many cases extraction of teeth is the treatment necessary, and I am on record in your columns to this effect (1906). Apart, however, from differences of opinion in the interpretation of radiograms as to actual conditions present and the treatment thereof, a great divergence seems to have arisen in connection with "preventive treatment," and one line of argument seems to be as follows: Gingivitis is an early stage of pyorrhoea. If the patient has no pyorrhoea now so much the better, but he must take no risks and lose his teeth lest a worse thing come upon him. It is further urged that by removing the teeth whilst the alveolus is healthy, the latter will not be absorbed and will afford good support for artificial substitutes. But the "extractors" are not taking themselves alone along the easy way; unfortunately, impressionable students are being taught the steps. I have in mind a candidate for the L.D.S. diploma who, having examined a case of inferior protrusion in a young girl, was asked if he could suggest any treatment. He blithely replied that all the teeth should be removed. As soon as the examiner recovered he ventured to ask the reason for such a decision, and received the reply that a probe could be passed between the teeth and the gums. The word "millimetre" was used, perhaps to give the impression of scientific accuracy, but the candidate appeared to be honest in his belief. Sir James Goodhart says that "an exaggerated view is too frequently taken of trivial conditions of the buccal mucous membrane." May I quote in confirmation one of the most striking cases I have recently seen. A young medical man, having a history of some abdominal trouble, said that a provincial dentist had condemned him to lose all his teeth. The patient had allowed two to be extracted, and had been sent to town for a second opinion, taking with him the now fashionable sheaf of radiograms. These did not appear to throw much light (or shade) on the matter, but the verdict was confirmed. When I saw him later there was no trace of pyorrhoea; there was no recession of gums; they were of a healthy pink colour with the exception of a very slight gingivitis localized to one lower incisor. There was no caries, the teeth were quite firm, and I hope that he has them still. Many will agree with Sir James Goodhart "that a harmful practice has come into vogue that needs to be held up," and will welcome his assistance.

A highly-qualified medical practitioner, Dr. Louis Stamm, of Streatham, also, in the course of a temperate letter, adds the following protest:—

When oral sepsis was first suggested years ago as a casual factor in many conditions of ill-health, one remembers with some gratification cases in which one was able by examination of the mouth and condemnation of the teeth "to put the finger on the spot" and clear up the trouble. But now the tables are turned and the patient comes with the diagnosis from the dentist: "The dentist says the cause of my anaemia (or whatever the ailment may be) is my teeth, and they must all come out." And, as Sir James Goodhart points out, when one comes to examine the mouth one finds in many cases a trivial gingivitis, often quite lacalized, which readily yields to treatment.

These conditions have assumed such a magnitude of importance in the professional mind as casual factors of disease that their relative position as *results* of ill-health appear to be entirely overlooked. But actually the oral affection is the result of a depressed vitality from some other cause in many more cases than it is the primal cause of the disease. In such cases, leaving aside the brutal mutilation involved, extensive extraction of teeth often has a most disastrous effect upon the already depressed vitality of the patient.

It is incumbent upon the leaders of the profession, and especially of the dental profession, to take steps to bring about a more steady mental balance on this question, for, as Sir James Goodhart says, a good many of us have lost our heads, and unfortunately when we lose our heads, it is the innocent, ignorant, trustful public who suffer.

---

Our colleague, Mr. William Rushton, turning the tables against the medical fraternity as most responsible for "pyorrhea panic," says:—

Many dental surgeons will thank Sir James Goodhart for uttering a protest against the wholesale extraction of teeth now so prevalent. It is perfectly true that, as he says, teeth are "pounced upon" as the cause of many disorders, including cancer, and it is a matter of great difficulty to persuade patients that they have not got pyorrhea when their medical attendant assures them that they have. When we hear of aged persons going through the severe operation of multiple extractions from which they do not always recover, and of others who have bartered their powers of mastication and speech and altered their features for a problematical benefit, it is time somebody spoke out. At the same time the other extreme must be guarded against—that of preserving and scaffolding up loose and septic teeth.

---

Again, an experienced surgeon, Mr. G. Sherman Bigg, acknowledging the responsibility of the medical profession for much "exaggeration of diagnosis," continues:—

Sir James F. Goodhart's protest against the extraction of teeth for so-called pyorrhea alveolaris and his criticism of the routine practice by vaccine treatment are the expression of the thoughts of many members of the medical profession. Recently a patient of mine had most (why not all?) of his teeth removed because he was said to be suffering with "aortic arteritis," as a matter of fact, from simple dyspepsia, the result of over-eating and insufficiency of exercise. Without his teeth he could only eat small quantities of soft food, and within a fortnight the aortic arteritis had ceased to be in existence.

Seriously, this craze of fashion in diagnosis is detrimental to the prestige of the medical profession. It cannot be to our advantage that the diagnosis made by one man should be condemned as absolutely wrong by another member of the same profession, and yet it is almost impossible to avoid doing so, and tell the truth. This last week, when I was endeavoring to minimize my difference of opinion, the patient said to me, "Please give a name to my disease," and what could one do under the circumstances?

---

This was followed by a weighty protest from Mr. R. Denison Pedley, who in the course of his letter said:—

We who spend the best part of our professional hours in trying to save teeth are often in the anomalous position of conflict with both physicians and surgeons. This is due to the fact that we are daily requested to sacrifice sound teeth because of some slight marginal inflammation or retrocession of the gums, misnamed pyorrhea alveolaris. We are well aware that time will correct this mistaken zeal, for there is an innate desire to keep what Nature has supplied, and "the fashion of this world passeth away." Meanwhile, it needs the words of a wise physician to call a halt, for this needless sacrifice is spreading far and wide, even to the children.

---

In the following issue of the *Lancet* (August 8, 1914,) Mr. J. G. Turner, as apologist for the "extractors," writs:—

May I put my finger at once on the weak spot of Sir James Goodhart's protest in the *Lancet* of July 25 against "wholesale" extraction of teeth? It lies in the sentence, "I examined his mouth very carefully, and his teeth were to my eye perfect." It is exactly the failure on the part both of doctor and dentist to realize on the one hand the amount of dental sepsis, and on the other the extent to which the tissues resent its presence— *i. e.*, the local signs of inflammation and destruction or hypertrophy of tissue—that leads to the present conflict of opinion. Sir James Goodhart asserts that "pyorrhea" is a painful malady; pain is an uncertain symptom, often absent through the whole course of the disease. His description of "pyorrhea" as deep-seated, pent-up suppuration in the alveoli does not apply to the great majority of cases met with in dental practice. Pent-up pus means acute abscess and pain, but the usual case of "pyorrhea" presents at the necks of the teeth freely discharging sinuses; if a sinus become blocked, then there is the acute state Sir James Goodhart refers to. He also speaks of "trivial ulceration of the edges of the gums." The ulceration or "pyorrhea" is not found patent on the edges of the gums; it is hidden on the tooth-ward side of the gum-flaps that help to form the pockets of "pyorrhea". Perhaps I may be excused for suggesting that Sir James Goodhart's protest may not be so well founded as at first sight it seems.

Till lately teeth were preserved at all costs, and perhaps the swing of the pendulum has in some individual cases gone too far. I suspect we could all relate cases in which we have saved the patient's teeth after "wholesale" extraction has been advised, but my daily experience convinces me that in the gross more good is done by "wholesale" extraction of teeth that are suspect than by conservation.

---

Mr. Turner is supported by Mr. F. St. J. Steadman as follows: "It is very clear from Sir James Goodhart's letter that he has not devoted the special attention necessary to make himself acquainted with the methods of diagnosing periodontal disease. Had he taken the trouble to wrap a whisp of cotton-wool round a very thin bristle and gently passed this round each tooth in the case he mentions he would probably have found "pockets" round the condemned teeth. Then had he warmed the bristle after withdrawing it from these pockets and held it under his nose he might possibly have had some glimmering as to the justice of the condemnation. Further, had he had a skiagraph taken to show the condition of the alveolar processes he would probably have seen areas of rarefying osteitis an possibly even abscess cavities round some of the roots."

---

As a rejoinder, Mr. George Thompson says: I desire as a dental surgeon to a London hospital to support Sir James F. Goodhart's contention in the *Lancet* of July 25, that " ɪ harmful practice has come into vogue that needs to be held up." Extreme methods are usually wrong. I have to see a great many dirty mouths in which there is no deep-seated suppuration, and my experience confirms the view that scaling of the teeth and the use of the tooth-brush twice daily with a corrected dietary yield quite marvellous results. The term pyorrhœa has been used too often loosely to cover all conditions of oral sepsis.

A condition of asepsis is impossible in any mouth, therefore the term is relative. Teeth that are firm enough to be used in mastication should be saved. It is quite easy to remove all the teeth, but not so easy to restore the function of mastication, with an abundant flow of saliva and all the values of trituration of foods in the mouth.

---

A medical contribution to the controversy was made in the *Lancet* of August 22 by Mr. C. Knox Bond, of Builth Wells, who states that:—

A patient, a lady of a lifetime of imaginary disorders, drew my attention to the fact that she had undergone extraction of her entire set of teeth since I had seen her a year previously. On further inquiry she informed me that her dentist, whom she had consulted for some trivial dental trouble, had informed her she was suffering from "Rigg's disease," and that it would be necessary to extract all her teeth. Her imaginary ailment at the moment was colitis; this she was assured was caused by "Rigg's disease," and in the event, and with the sanction of her medical attendant, she was rendered edentulous. She assured me with pride that her teeth when drawn were perfectly sound, and I have no doubt they were.

The impression made on my mind at the time was that I had never in a lengthy and varied experience seen so cruel and unwarrantable a procedure under the guise of treatment. In the name of a disease which was not present she was subjected to this outrage for the relief of a condition which was non-existent. It is to be hoped now that the subject has been called attention to under the authority of Sir James Goodhart's name that this form of malpraxis may cease.

---

The whole correspondence was replied to by Dr. Goodhart on August 22, by a letter wherein he says: "I do not think it necessary, or even desirable, to make any detailed reply either to Mr. J. G. Turner or Mr. F. St. J. Steadman, who alone have criticized my letter adversely. But I would like to say this to make my exact position clear—that each of these gentlemen seems to me to hold that pyorrhea is a common disease, an insidious one, easily overlooked, yet associated with definite changes in the bones, and that it leads to septic intoxication. If what I in my ignorance call gingivitis they call pyorrhea alveolaris, so be it. My essential point is, however, is it the cause of septic absorption, and is it worthy of the extraction of the teeth in bulk?

Now I will admit Mr. Steadman's impeachment that I have no pretension to being an expert in dentistry, but I am already too familiar with the term 'pockets' which both he and Mr. Turner speak of, and I am always on the look-out for them in my poor non-expert way, and I should suppose that anything worthy of such a description should contain pus, exuding under pressure such as the eye could see. Yet neither to my sight nor to pressure of the finger along the gums have I been able to detect this in a considerable number of cases. I certainly 'have not taken the trouble to wrap a whisp of cotton-wool round a very thin bristle, and gently pass it round each tooth to find them,' nor have I 'warmed the bristle after withdrawing it, and held it under my nose.' I am quite sure that the teeth of very few of us would stand that test; indeed, if that be my critic's criterion of pyorrhea alveolaris I can only suppose that he has already immolated all his own ivories and been exceptionally in the set 'made-at-homes' with which he has supplied himself. To one not an expert it seems more reasonable to hold that so long as teeth are good, firm in their sockets, free from pain, and serviceable for easy mastication, there is something to be said on the side of keeping our own rather than taking to others that we know not of. We seem to be far too ready to jump from the latest hypothesis of oral sepsis to the proof thereof. The changes said to occur in the bone in these cases, for example, have they been clearly distinguished from those that must exist in company with retrocession of the gum?—an exceedingly common condition that carries with it usually no sign of sepsis.

Again, what adequate evidence is there to prove that 'the ulceration of pyorrhea not found patent on the edges of the gums, but hidden on the toothward side of the gum-flaps, that help to form the pockets of pyorrhea'—if it exist—'frequently gives rise to gross joint lesions, and many other maladies too numerous to mention.' I do not deny it; all I ask for is evidence. Effects from certain causes are too readily assumed. When first the oral sepsis explanation of pernicious anaemia was announced there was the usual hue and cry. How far has it been substantiated? I have seen many a case of pernicious anaemia since then. I cannot record one—I had seen one such before—where attention to the teeth has had any certain effect upon the disease, and it can be but seldom that the suggestion can have helped to its cure."—*British Dental Journal.*

